# O.6.2-3 The Global Observatory for Physical Education (GoPE!): monitoring physical education and physical activity in children and adolescents worldwide

**DOI:** 10.1093/eurpub/ckad133.273

**Published:** 2023-09-11

**Authors:** João Martins, Marcos Onofre, Rafael Cristão, Andrea Ramirez Varela, Michael Pratt, Pedro C Hallal

**Affiliations:** Faculdade de Motricidade Humana e UIDEF, Instituto de Educação, Universidade de Lisboa, Lisboa, Portugal; Faculdade de Motricidade Humana e UIDEF, Instituto de Educação, Universidade de Lisboa, Lisboa, Portugal; Faculdade de Motricidade Humana e UIDEF, Instituto de Educação, Universidade de Lisboa, Lisboa, Portugal; School of Medicine, Universidad de los Andes, Bogotá, Colombia; Institute of Public Health, Herbert Wertheim School of Public Health & Human Longevity Science University of California San Diego, United States; Department of Kinesiology and Community Health, University of Illinois Urbana-Champaign, United States; jmartins@fmh.ulisboa.pt

## Abstract

**Purpose:**

It is estimated that 80% of children and adolescents do not meet the 60 min of moderate-to-vigorous intensity physical activity (PA) per day to benefit their health.^1,2^ Strengthening the provision of quality physical education (PE) and PA experiences within schools is a highly recommended policy action.^3^ Furthermore, there is a gap in monitoring systems of PE&PA at schools, which would contribute to tracking progress and inform decision-making to drive PE&PA policy, action, and research in this population group.^4^ After 10 years of successful monitoring of adults’ PA by The Global Observatory of Physical Activity (GoPA!), there is an urgent need to complement and initiate high-quality monitoring in children and adolescents. The Global Observatory of Physical Education (GoPE!) will be launched in 2023, therefore, our purpose is to present the GoPE! to the HEPA network.

**Project description:**

Placed within the GoPA!, GoPE!’s main mission is to monitor PE and PA in children and adolescents worldwide. The GoPE! monitoring system will use a set of surveillance, policy, and research standardized indicators to allow countries to initiate, determine and improve their current status and progress around PE and PA. Standardized indicators will be updated every 3-4 years, allowing comparability between countries and world regions. GoPE! will publish the country profiles [GoPE! Country Cards], which are going to be developed following the GoPA! stepwise approach, and will consolidate as an independent evidence and local representatives-based and global surveillance system including the best PE&PA and sports experts, researchers, practitioners, and policymakers.

**Conclusions:**

GoPE! will demonstrate its critical role in facilitating periodic PE&PA surveillance, policy and research data, to inform promotion, advocacy, and agenda-setting efforts to achieve international goals and recommendations. GoPE! and GoPA! will continue to monitor progress to battle the global pandemic of physical inactivity across the life course.

**References:**

^1^ USDHHS. *2018 PA Guidelines Advisory Committee Scientific Report.* Washington: USDHHSD;2018.

^2^ van Sluijs, E. et al. PA behaviours in adolescence: current evidence and opportunities for intervention. *The Lancet.* 2021;*398*(10298):429-442.

^3^ WHO. GAPPA 2018–2030: more active people for a healthier world. Geneva;2018.

^4^ WHO. Global status report on PA 2022. Geneva: WHO;2022.

